# Tea (*Camellia sinensis*) infusions ameliorate cancer in *4TI* metastatic breast cancer model

**DOI:** 10.1186/s12906-017-1683-6

**Published:** 2017-04-07

**Authors:** Karori S. Mbuthia, Paul O. Mireji, Raphael M. Ngure, Francesca Stomeo, Martina Kyallo, Chalo Muoki, Francis N. Wachira

**Affiliations:** 1 Department of Biochemistry and Molecular Biology, Egerton University, P.O Box 536 20115, Egerton, Kenya; 2 Department of Epidemiology of Microbial Diseases, Yale School of Public Health, 607 Laboratory of Epidemiology and Public Health, 60 College St, New Haven, CT 06510 USA; 3 Department of Veterinary Clinical Studies, Egerton University, P.O Box 536-20115, Egerton, Kenya; 4 Biosciences eastern and central Africa- International Livestock Research Institute, (BecA-ILRI) Hub, P.O Box 30709-00100, Nairobi, Kenya; 5 Crop Improvement and Management Programme, Kenya Agricultural and Livestock Organisation-Tea Research Institute (KARLO-TRI), P.O Box 820-20200, Kericho, Kenya; 6 South Eastern Kenya University, P.O Box 170-90200, Kitui, Kenya

**Keywords:** Catechins, Egcg, Theaflavins, Anthocyanins, Antioxidant activity, Caspases

## Abstract

**Background:**

Tea (*Camellia sinensis*) infusions are widely consumed beverages with numerous health benefits. However, physiological and molecular responses mediating these activities are poorly understood.

**Method:**

Three replicates of *4TI* cancer cell suspension (2.0 × 10^5^ cells/ml) were challenged in vitro with various concentrations of green, black and purple tea infusions to asseses their cytoxicity and associated differentially expressed genes in the cells. Inhibitory activity was tested by using serial dilutions of respective tea infusions in a 96 well ELISA plate.

**Results:**

Green tea had the highest inhibition on *4TI* cells proliferation at a concentration of IC_50_ = 13.12 μg/ml. Further analysis of the *4TI* cancer cell line treated with tea using 454 pyrosequencing generated 425,696 reads with an input mean length of 286.54. Trimmed sequences were imported on a CLC genomic workbench v7.03 and annotated on a reference mouse genome (*Mus musculus* strain *C57BL/6 J*). Results revealed a differential expression of apoptosis related genes in the transcriptome. *Casp8, Casp9, Casp3, Casp6, Casp8AP2, Aifm1, Aifm2* and *Apopt1* genes were significantly upregulated indicating the process of apoptosis was initiated and executed.

**Conclusion:**

These findings on caspases offer valuable information on the mechanism of tea as an anticancer agent and will contribute to further research in future novel treatments.

## Background

Tea, from (*Camellia sinensis* L.O Kuntze) is a widely consumed beverage in the world. Phytochemicals and functional components in tea are receiving a lot of attention due to their potential benefits in health when consumed as part of a varied diet on a regular basis and at effective levels. Many nutraceuticals, functional foods and naturally occurring polyphenols have physiological and pharmacological activities including their well characterized antioxidant properties [1–3]. The scientific and food industry communities share a common goal of extending the quality of human life by utilizing phytochemicals. The potential start point in this is through development of viable options for the management of chronic diseases through the use of nutraceuticals and functional foods. This is because functional foods are fairly affordable, readily available and extremely active, have profound effect on cell metabolism and often demonstrate few side effects [4]. It is evident that nutraceuticals offer a selective advantage over synthetic drugs necessitating need to investigate their usefulness to human health.

Tea is rich in polyphenols associated with numerous pharmacological properties such as; antimicrobial [5], anti-inflammatory [6], anti-aging [7, 8] antimalarial [9] and antioxidant activity [1, 3, 10]. Cancer, accelerated aging and other chronic disease associated with free radical induced oxidative damage [4] can be ameliorated by phytochemicals in tea since antioxidants in tea have been shown to protect DNA from damage induced by free radicals and slow or halt initiation and progression of cancerous tumor growth [12]. Epicatechin gallate (EGCG) a polyphenol in tea, has been shown to inhibit growth of cancer cells as well as play an important role in stimulating apoptosis or programmed cell death both of which are crucial aspects of cancer prevention [13, 14]. Biochemical actions of green tea and mainly the catechin EGCG in chemoprevention and anticancer effect have been studied by various reseachers [15–20]. Further, there is also increasing evidence that tea provides significant immuno-protective qualities to cancer patients undergoing radiation and chemotherapy [19]. However, mechanisms of action of anti-cancer properties are poorly understood. Additionally, published data have primarily focused on green tea yet black tea is the most widely consumed tea product worldwide. Data on purple tea which is a novel product rich in anthocaynins is also lacking.

This study set out to establish possible mechanisms of cancer prevention by Kenyan green, black and purple teas using the *4TI* metastatic breast cancer model. Gene expression profiles of the *4TI* cancer cell line were analyzed using the 454 pyrosequencer technique; a fast, simple, and cost effective method of determining candidate genes involved in major metabolic pathways. The technology also provides a more comprehensive and efficient way to measure transcriptome composition and obtain RNA expression profiles [21]. In addition, whole-genome sequencing and analysis of cancer and matched normal genomes with next-generation sequencing platforms can illuminate commonly mutated genes and transcript-level events that contribute to the underlying cancer biology.

## Methods

### Test tea and cell culture samples

Test tea samples were sourced from farms of the Tea Research Institute (TRI) (0°22′18.54″S, 35°21′3.19″E, 2180 m asl) in Kericho Kenya where tea is commercially grown. The tea samples were obtained from fresh leaves consisting youngest two leaves and a bud from tea bushes. These samples were processed into black, green or purple tea and stored in alunimium foils as detailed in Karori, et al. [2] for use in analysis. Cell cultures (*4TI* metastatic breast cancer cells) were obtained from the Center for Traditional Medicine and Drug Research (CTMDR), Kenya Medical Research Institute (KEMRI), Nairobi, Kenya in cryopreserved form. The 4TI metastatic breast cancer cells were maintained at pH 7.2 in Minimal Essential Medium (MEM) (Invitrogen, Carlsbad, CA, USA) supplemented with 2 mM L-glutamine, 5% fetal calf serum (FCS) (Sigma, USA) and 2% penicillin-streptomycin at 37 °C in humified incubator at 5% CO2. Cells were grown in standard T75 tissue culture flasks and upon reaching 80% confluence were passaged with a solution of 0.25% trypsin-ethylene di amine tetra acetic acid (EDTA). The cells were cultured for a maximum of 10 passages after retrieval from the fresh cells in the frozen stock.

Approval to conduct research on the 4TI cells culture was obtained from KEMRI Institutional Ethical Review Committee (protocol number SCC 2435) and National Institute of Health (approval number 991073).

### Assessment of tea infusions effects on growth and proliferation of *4 T1* cells

Effects of black, green or purple tea infusions on viability and proliferation of the *4 T1* cells was determined using 3-[4, 5-dimethylthiazol-2-yl]-2, 5-diphenyl tetrazoliumbromide (MTT) salt assay (Roche Diagnostics GmbH, Germany) as described in ATCC catalogue number 30-1010 K. Samples and reagents were distributed in a standard 96 wells plate in three independent replicates. Then each plate was incubated for 24 h to permit the cells to attach on surface of the respective wells in the plate. All media were subsequently replaced by 150 μl of black, green or purple tea (600 mg/ml) infusions in the last row (Row H) in each plate. In order to obtain a threefold dilution, serial dilution was carried out by removing 50 μl from wells of row H and adding to wells of row G. The contents were well mixed and 50 μl transferred from row F. This was continued upto row B where 50 μl was discarded so as to leave row A intact. Contents of the first row (row A) in each plate were exemepted from the serial dilution procedures and therefore acted as the negative controls for the wells challenged with respective tea infusions. Each plate was incubated at 37 °C, 5% CO_2_ for 48 h after which MTT (10 μl of 5 mg/ml stock solution in PBS was added into each well and were subsequently further incubated at 37 °C for 2 h. Resultant purple precipitates of formazan in each plate were dissolved in 150 μl of dimethyl sulfoxide (DMSO). Absorbances of the contents of the wells were recorded at 540 nm using Bio-Rad 3350 microplate reader (Bio-Rad, Hercules, CA, USA) according to the manufacturer’s instructions. The absorbances of the experimental wells were used as an index proxy for viability in relation to the readings absorbances of the control wells that were representatives of 100% viability.

### Statistical analysis

Data obtained were expressed as mean ± SEM of replicates and analyzed using the Alamarblue assay for IC_50_. Comparison between data sets was performed using one way analysis of variance (ANOVA) followed by Student’s *t*-test. All statistical analyses were performed using GraphPad Prism 5.0 (GraphPad Software Inc., San Diego, CA). Differences were accepted as statistically significant at *p* < 0.05.

### Cell harvesting for mRNA isolation

Cells at a density of 2.0 × 10^5^ cells/ml were cultured in T75 flask and allowed to attach for 24 h and at 70% confluent, the cells were treated using green, black or purple tea extracts at the respective IC_50_ obtained above. The cultures were incubated at 37 °C in a humified incubator at 5% CO_2._ After 24 h, the cells were then observed under a microscope for morphological changes.

The media from the plate was discarded and 2xPBS added to the flask for washing. A volume of 1 ml trypsin was added to the flasks and cells incubated for 2 min to allow for detachment. Soon after, the suspension was pipetted into eppendorf tubes and centrifuged (Eppendorff centrifuge 5810R, A-4-62 rotor) at 15000 rpm for 10 min to remove the supernatant. The cells were washed in 1xPBS and centrifuged again at 1500 rpm for 3 min. New PBS solution was then added to cover the cells; and the cells were then frozen at -196 °C in liquid nitrogen for mRNA isolation.

### Extraction and quantification of mRNA

Extraction and quantification of mRNA was carried out at the BecA-ILRI Hub laboratories after an ethical approval by ILRI Institutional Research Ethics Committee (IREC) and given protocol number IREC2013–05. Isolation of mRNA was carried out using mRNA isolation kit Catalogue Number 11741985001 (Roche, Germany) but with slight modification. In addition, reference was made on work by Michaela, et al. [22] on the utilization of paramagnetic particles. A cell density of 1.0 × 10^7^ cells/ml was used as the starting material. The cells were washed three times in ice cold PBS and spun at 15000 rpm for 3 min at 4 °C. A volume of 1 ml of lysis buffer was added to the cells and vortexed for 15 s. DNA was mechanically sheared by passing the cells 10 times through a 21 gauge needle. Streptavidin-coated–magnetic particles (SMPs) were re-suspended thoroughly and 100 μl pipetted into fresh micro tubes. The SMPs were immobilized on the side of the container with a magnetic particle separator. The entire storage buffer was removed and SMPs re-suspended in 500 μl of lysis buffer. The entire lysis buffer was removed after immobilizing the SMPs with a magnetic particle separator. A volume of 6 μl of Biotin-labeled Oligo(dT) was added to the sample and mixed well to form a hybridization mix. After a careful mixing, the hybridization mix was added to the tube containing the already prepared SMPs and re-suspended. The mixture was incubated at 0 °C for 5 min followed by the separation of SMPs from the fluid for 30 min. The SMPs were washed in 500 μl of wash buffer 5 times then the wash buffer quantitatively discarded after the last washing step. mRNA was eluted by adding 50 μl of double distilled water to the SMPs and incubating for 2 min at 65 °C. The mRNA was transferred to fresh define (RNase)-free tubes and stored at -80 °C.

Purity and concentration of mRNA was assessed by determining the absorbance of the sample at 260/280 nm using a 2000-NanoDrop spectrophotometer (Thermo Fisher Scientific-USA). The quality of the extracted mRNA was also checked by electrophoresing it on a 2% agarose gel. The samples were denatured by adding 5 μl of RNA sample buffer (95% deionized formamide, 5 mM EDTA, 0.025 xylene cyanol FF, 0.025% bromophenolblue and 0.025% sodium dodecyl sulphate (SDS). The samples were then put in a water bath at 65 °C for 5 min and immediately immersed in ice after the lapse of 5 min to avoid renaturation. The total mixture was loaded on a 2% agarose gel stained with 2.5 μl of (10,000×) GelRed and electrophoresed for 25 min at 100 V.

### Preparation of cDNA library for Transcriptome sequencing

The cDNA library was synthesized using cDNA rapid library preparation kit (GS FLX Titanium Series- 2010 Revised Edition, Roche (Germany) according to manufacturer’s instruction. The sample mRNA (200 ng) was fragmented into smaller pieces at 70 °C for 30 s in the fragmentation buffer (0.1363 g of ZnCl_2_, 1 M Tris-HCL pH 7.0 and 1-10 ml of molecular grade water) and denatured by adding Roche primers ‘random’ for 10 min at 70 °C. A volume of 2 μl of 0.5 M EDTA at a pH of 8.0 and 28 μl of 10 mM Tris HCL were added and mixed with 80 μl of RNA clean reagent containing solid phase reversible immobilization (SPRI) beads. The mixture was mixed well at room temperature for 10 min then separated using a 96 ring magnetic particle separator (MPC). The beads were washed carefully using 200 μl of 70% ethanol and the supernatant carefully discarded.

To the fragmented RNA, 4 μl of the random Roche primer was added and vortexed for 10 s. With the tube in ice, the fragments were reverse-transcribed to synthesize first strand cDNA using avian myeloblastosisvirus (AMV) reverse transcriptase (Roche, Germany) -μl of 5xRT-buffer AMV, 4 μl of 0.1 M DTT, 4 μl of 10 mM dNTPS, 1 μl of 25 U/μl protector RNase inhibitor and 2 μl of 25 U/μl AMV RT). Subsequently, the second strand cDNA was synthesized using second strand enzyme (Roche- 30 μl of 5 × 2 buffer, 72 μl redistilled water, 1.5 μl of 10 mM dNTPs and 6.5 μl of a second strand enzyme). The mixture was spun for 2 s and incubated at 16 °C for 2 h before adding 20μlof T4 DNA polymerase (Roche, Germany). The 1.7 ml contents were transferred to a 2.0mlmicrocentrifuge and 300 μl of AMPure beads added. The beads were separated using a MPC and washed carefully using 70% ethanol. The beads were allowed to air dry at room temperature for 3 min, washed using 16 μl of 10 mM Tris-HCL, allowed to pellet and the pellets containing the ds-cDNA transferred to a fresh 200 μl PCR tube.

The cDNA fragments were end repaired using an end repair mix composed of 1 μl T4 DNA polymerase, 1μlTaq polymerase and 2.5 μl of polynucleotide kinase buffer, 2.5 μl of ATP, 1 μl of dTNP) before ligation of adaptors with multiplex identifier (MID) adaptor and DNA ligase (Roche, Germany) at 25 °C for 25 min. The products were purified to remove fragments less than 50 bp long using individual sample clean up (ISC) sizing solution (Roche, Germany). The cDNA library was then quantified and assessed for quality using TBS 380 Fluorometer(Turner Biosystems, USA) and Agilent bioanalyzer high sensitivity DNA chip (Agilent Technologies, Germany), respectively.

### EmulsionPCR amplification

The emulsion PCR (emPCR) amplification was done using the emPCR KitGS FLX Titanium series (Roche, Germany). In brief, the adapter containing quality cDNA was mixed with capture beads, PCR reagents and emulsion oil and run using the following program;1 × 94 °C for 4 min, 50 × 94 °C for 30 s, 58 °C for 4.5 min, 68 °C for 30 s, 10 °C on hold. After the PCR reaction the beads were checked for emulsion breakage which is a distinct layer with a clear middle layer. The broken emulsion were discarded while intact emulsion were used for bead recovery. Those beads that did not hold DNA were eliminated while beads holding more than one type of DNA were recovered and washed. To ensure that only the beads carrying the amplified DNA was used in the sequencing, the recovered beads were enriched by hybridization of the biotinylated enrichment primer to the adaptor of each amplified DNA template to which it is complemetary in its binding to streptavidin-coated magnetic beads. The beads carrying the amplified DNA were separated from the null and poorly amplified beads using a magentic particle separator. The DNA library beads were separated from the magnetic beads by melting the amplification products away from the enrichment primer, leaving a population of bead-bound single-stranded template DNA fragments which are immobilized and amplified DNA library. The final step in the emPCR amplification process was the annealing of sequencing primer to the amplified DNA template to form a library of clonally amplified DNA fragments ready for loading onto a picoTiter plate and sequencing.

### 454 sequencing

Sequencing was done from both the 5′ and 3′ end using 454 sequencing technology at the BecA-ILRI Hub for the tea treated groups and the control group. Sequencing was accomplished using a GS FLX Titanium sequencing kit (Roche, Germany) according to the manufacturers’ instruction. In brief, the DNA-capture beads were loaded onto PicoTiterPlate™ such that each well contained single DNA beads. Sequencing reagents comprising of deoxynucleotide triphosphate(dNTP) buffers, sodium chlorite tablet, Apyrase, Ppiase, inhibitor TW reagent, enzyme beads, bleach and DTT were sequentially flowed over the plate. The sequencer automatically performed and monitored the sequencing reactions in all the wells of the PicoTiterPlate simultaneously. The raw output of a sequencing process consisted of a set of digital images (PIF files) from which the sequence of the DNA library fragments was read. The processing of images to sequences and base-calling calculations were all performed by the 454 data processing pipeline in which raw reads were obtained.

## Sequence analysis

### Quality control and de novo assembly of sequencing reads

Individual sequences from each treatment were extracted and separated from the raw 454 reads ssf file based on their unique multiplex Identifier (MID) sequence tag using sfffile and converted into their respective fastq files using sffinfo routines in Newbler v2.3 (Roche GS De Novo Assembler v2.3; Roche Life Sciences, Inc.). Before assembly, the raw reads were filtered using FASTX-tool kit to obtain the high-quality clean reads by removing adaptor sequences, duplication sequences, the reads containing more than 10% “N” rate (the “N” character representing ambiguous bases in reads), and low-quality reads with minimum length of 50 withquality score cut-off (Q-value <20) and minimum percentage of 75 (p75). The Q-value is the quality score assigned to each base and was defined by the equation;

Q =  − 10log_10_(e);

Where e is the probability of a base call being wrong. The higher the score, the lower the probability of a wrong base call during sequencing.

The sequences were again checked for data quality using FASTQC (Babraham Institute, Cambridge, UK) and imported into the CLC Genomics Workbench v7.03 software (CLC bio, Aarhus, Denmark) for post-processing and data analysis. Briefly, the sequences were trimmed based on the FASTQC report and were mapped onto annotated mouse genes with support from reference mouse (*Mus musculus* strain C57BL/6 J) genome and transcript/mRNA from mouse using RNA-Seq routine. All the mouse genome and annotations were obtained from the mouse genome project (sanger.ac.uk/resources/mouse/genomes). Using the trancripromic analysis routine in the software, the data were normalized by total reads per million and analyzed for differential gene expression empirical analysis of differential gene expression subroutine based on reads per kilobase of transcript per million reads mapped (RPKM). Promising up and down regulated candidate genes were selected on basis of a combination of their RMPM, *P-*values and folds change values.

## Results and discussion

### *4TI* cell culture assay

**Fig. 1:**
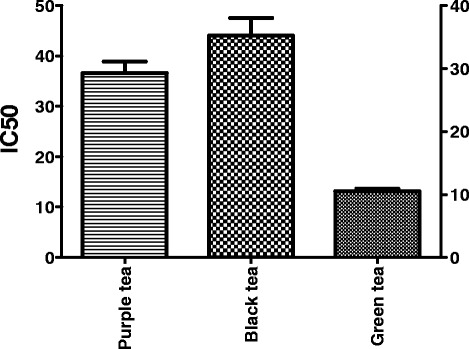
Quantification of the inhibitory concentration (IC_50_ μg/ml) of purple, black and green teas on *4TI*cancer cells

The reduction in the viability and proliferation of *4T1*cancer cells were determined using the3-(4, 5-di methyl thiazol-2-yl)-2, 5-diphenyltetrazolium bromide) (MTT) assay. Results obtained in this in vitro toxicity assay were analyzed and results presented in Fig. 1. Green tea had the highest inhibition on cancer *4TI* cells at a concentration of IC_50_ = 13.12 μg/ml, black tea the lowest at IC_50_ = 44.04 μg/ml and purple tea had an inhibition of IC_50_ = 29.27 μg/ml. The inhibition on *4TI* cells by the teas analysed in this study was significantly different (*p* ≤ 0.05).

Cancer cell concentration and viability in the presence of the tea extracts were compared with that of the control and the definition of cytotoxicity described by Malebo, et al. [23] and Zofou, et al. [24]. The definition applied in this study to rate cytotoxicity was as follows; IC_50_ < 1.0 μg/ml (high toxicity); IC_50_ of 1-10 μg/ml (moderate toxicity): IC_50_ of 10-30 μg/ml (mild toxicity) and an IC_50_ > 30 μg/ml was considered nontoxic. Based on this, green and purple teas had a mild toxicity whereas black tea had no toxicity towards the cells (Table 1).

**Table 1:** Cytotoxicity of various tea extract

	Tea samples		
	Green tea	Black tea	Purple tea
IC_50_ (μg/ml)	12.55	50.66	32.96
	14.25	38.49	28.13
	12.56	42.96	26.73
Mean (μg/ml)	13.12	44.04	29.27
Toxicity level	Mild	Non toxic	Mild
n	3	3	3
CV	7.46%	13.98%	11.17%

**Fig. 2:**
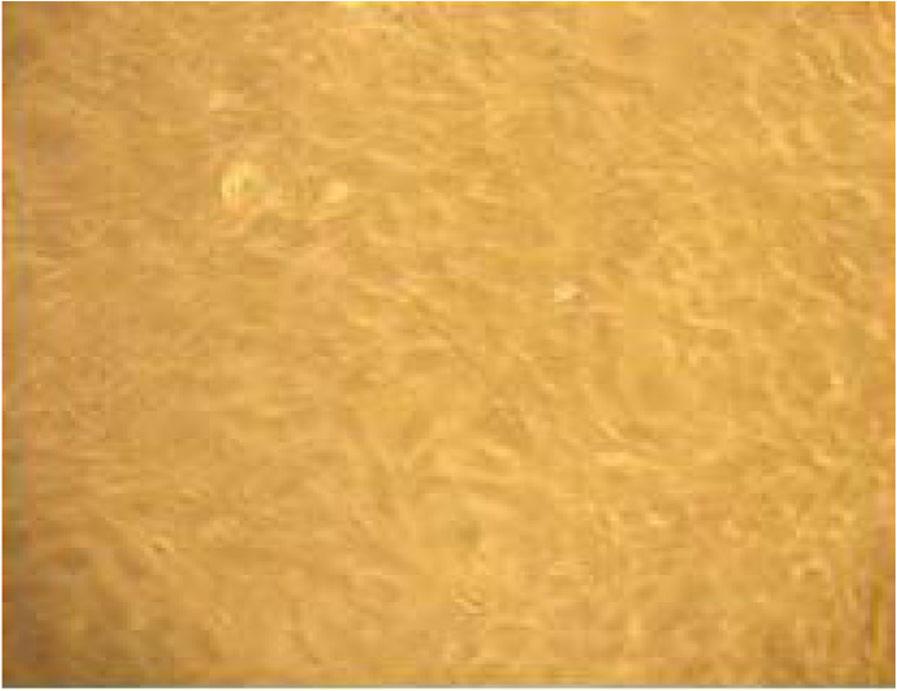
A monolayer of *4TI* control cells after 48 h in DMEM media

Based on the 3-(4, 5-di methyl thiazol-2-yl)-2, 5-diphenyltetrazolium (MTT) observation, the cells were cultured in a T75 flask to score the effect of various teas on cell growth. Results presented in Fig. 2 below clearly shows that the *4TI* cancer control cells after incubating using 5% CO_2_ for 24 h in Davis Minimal Essential media had their monolayer intact and the cells had attained a confluence of over 90%.

**Fig. 3:**
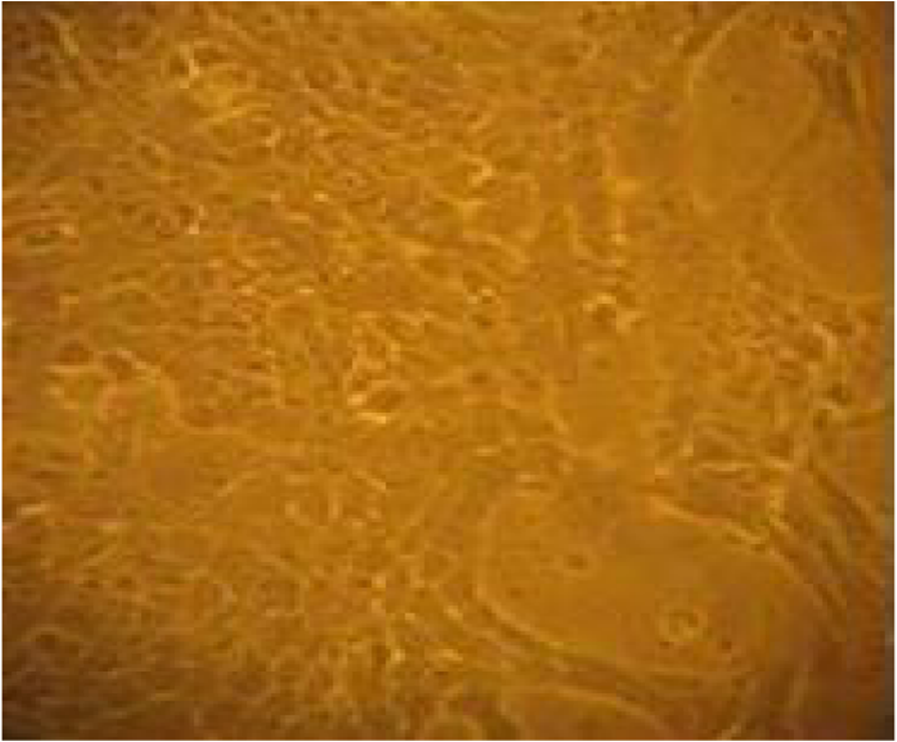
Sensitivity of *4TI* cancer cells to black tea at IC_50_ of 44.04 μg/ml showing disruption of monolayer

**Fig. 4:**
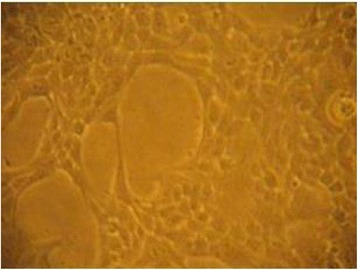
Sensitivity of *4TI* cancer cells to purple tea at IC_50_ of 29.27 μg/ml showing disruption of monolayer

**Fig. 5:**
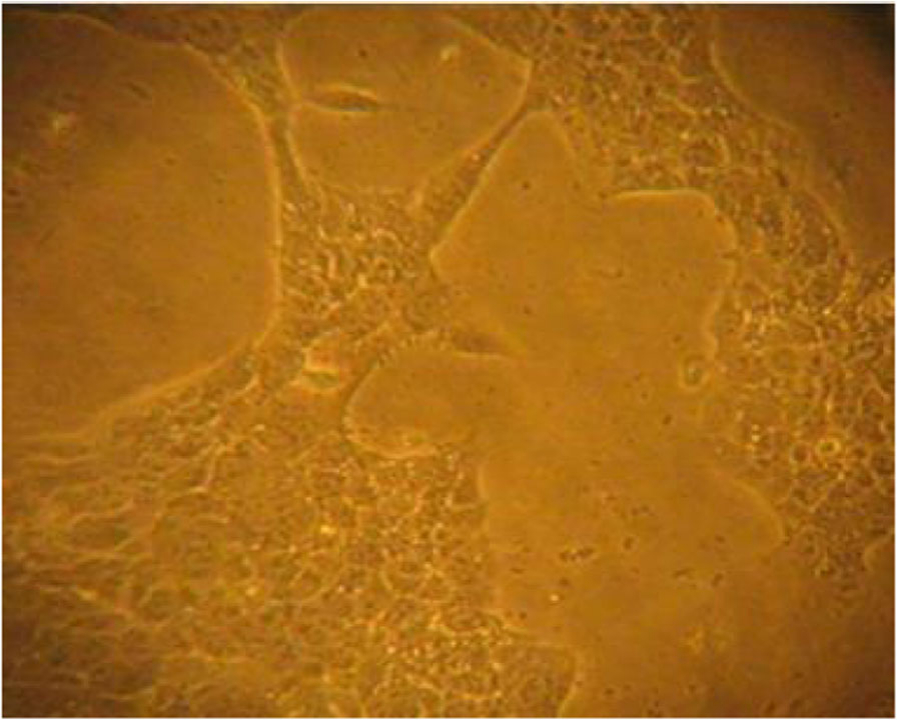
Sensitivity of *4TI* cancer cells to green tea at IC_50_ of 13.12 μg/ml showing disruption of monolayer

However, when the *4TI* cells were treated with black, purple andgreen tea an effect on the cell viability and proliferation was observed on the monolayer formation. Results from this study revealed that tea had an effect on cell growth by disrupting the monolayer formation which was evidenced by a reduction in the cell to cell contact and this was the reason why there was an ultimate reduction in the spreading of the cells (Figs. 3, 4 and 5). This remarkable observation was a clear indication that tea inhibited cell viability and proliferation.

Chemo-prevention agents with anticarcinogenic properties impact on cell populations by reducing, cell viability and proliferation. A reduction in cell proliferation rate indicates that there is a reduction in cell viability and the ensuing metabolic events that leads to apoptosis and necrosis. For the first time, this study attempted to determine the chemo-preventive effects of green tea, black tea and purple tea extracts in a highly metastatic breast cancer *4TI* cell line in vitro*.* In this assay the tea extracts caused a reduction in cell proliferation since the absorbance values of cells treated with tea extracts were lower than those of the control. This was caused by a reduction of the yellow tetrazolium salts by metabolically active cells in part due to the action of dehydrogenase enzymes to generate reducing equivalents such as NADH and NADPH.

Dysregulated proliferation in cells appears to be a hallmark of susceptibility to neoplasia and its regulation is critical in the prevention of cancer. The process of cancer prevention is generally associated with inhibition, reversion or retardation of cellular hyperproliferation and most flavonoids have been demonstrated to inhibit proliferation in many kinds of cultured human cancer cell lines [25, 26] with no similar cytotoxicity to human normal cells. Researchers have conducted in vitro bioassays on the potential anticancer activity of flavonoids in diverse cell systems. Hirano, et al. [27] examined anticancer efficacy of 28 flavonoids on human acute myeloid leukemia cell line HL-60, and compared differences between anti-proliferative activity and cytotoxicity of these compounds with those of four clinical anticancer agents. From his work, eight of the 28 flavonoids showed considerable suppressive effects on HL-60 cell growth with IC_50_ values ranging from 10 to 940 ng/ml. The lavonoid genistein had the strongest effects almost equivalent to the effects of current anticancer agents with little cytotoxicity against HL-60 cells, whereas the regular anticancer agents had potent cytotoxicity. Kuntz, et al. [25] screened more than 30 flavonoids to establish their effects on cell proliferation and potential cytotoxicity in human colon cancer cell lines Caco-2 and HT-29. He found out that nearly all compounds displayed anti-proliferative activity without cytotoxicity. Maricela and Walle, [26] examined the effects of quertecin (3, 5, 7, 3′, 4′-pentahydroxyflavone), a major dietary flavonoid, on cell growth and necrosis using oral squamous carcinoma SCC-9. Quercetin is the most abundant molecule in the extensive class of polyphenolic flavonoids ubiquitously found in plants and in many often- consumed foods, such as apples, onions, tea, berries, among others [28]. From their study, quercetin was initially shown to induce a stress response, resulting in necrosis of these oral epithelial cells. However, a prolonged exposure of the remaining cells to quercetin induced an apoptotic effect. The molecular properties underlying these effects were attributed to a wide array of biochemical mechanisms, including antioxidant properties [29] and effects on enzymes and signal transduction pathways involved in cell proliferation, cell cycle regulation and apoptosis [30].

The encouraging results of anticancer effects in preclinical studies have in the recent past stimulated the clinical trials of flavonoids in human. Wang, [31] extensively reviewed the therapeutic potential in human of four most widely investigated flavonoids namely flavopiridol, catechins, genistein and quercetin and found out that flavopiridol had achieved a proof-of-concept in phase I/IIa trials as a monotherapy. Mohbound and Khorshid, [32] studied the role of green tea polyphenols on the proliferation of human ovarian cancer cells in vitro and found out that green tea extract caused a reduction in cell proliferation. The inhibitory effect was attributed to the presence of 3–0-gallate group of the catechin structure which is a very efficient antioxidant [20]. Since green tea is rich in catechins, this explains why the activity of green tea in this study was higher than that of black and purple teas. During the processing of black tea, catechins as explained earlier are converted to highly polymerized substances namely TFs and TRs which have a lower antioxidant activity compared to green tea catechins.

Dietary flavonoids like those found in tea are natural antioxidants [1, 7, 11] that act by limiting the damaging oxidative reactions in cells, which may predispose cells to the development of cancer [33–35]. Oxygen-derived free radicals appear to possess the propensity to initiate as well as to promote carcinogenesis [36]. Chemically, flavonoids are capable of being one-electron donors and therefore serve as derivatives of conjugated ring structures and hydroxyl groups that have the potential to function as antioxidants in in vitro cell culture or cell free systems thereby scavenging superoxide anion, singlet oxygen, lipid peroxy-radicals and/or stabilizing free radicals involved in oxidative processes through hydrogenation or complexing with oxidizing species [37]. In vitro studies are able to demonstrate this activity for flavonols, flavones and most recently also for anthocyanins with the predominant anthocyanidin being malvidin [10, 38]. A correlation analysis between the different biochemical parameters in purple teas and antioxidant capacity has revealed that some anthocyanin/anthocyanidin fractions are individually and collectively potent anti-radical molecules. The molecules identified by Kerio, et al. [10, 38] included cyanidin- 3-O-galactoside, cyanidin-3-O-glucoside, peonidin and malvidin. Just like the catechins, the antioxidant efficiency of anthocyanins is related to several parameters which include the number of hydroxyl groups in the molecule, the catechol moiety in the B-ring, the oxoniumion structure in the C-ring, the hydroxylation and methylation pattern and to the acylation by phenolic acids [39]. Studies on the antioxidant activities of other beverages like wines have also revealed that glycosylated and methoxylated anthocyanins such as cyanidin-3-O-glucoside and malvidin-3-O-glucoside which are predominant in red wines are responsible for the antioxidant effect of red wine [40].

A study to determine the quenching of singlet molecular oxygen (1O_2_) by the flavylium cation revealed that the quenching efficiency was larger for the malvidin derivative, probably due to the electron donating methoxyl group in the B-ring of the malvidin molecule [40]. Further, it can also be deduced that the high antioxidant activities exhibited by the purple leaf coloured anthocyanin rich teas could be due to the catechin–anthocyanin complexes that are believed to have additional hydroxyl groups necessary for free radical-scavenging activity. This antioxidative activity of polyphenols found in purple tea can be used to explain why purple tea exhibited an activity against the *4TI* cancer cells.

Lee, et al. [41] used *4TI* cancer cells in vivo to explain why resveratrol (trans-3,4′,5-trihydroxystilbene) a naturally occurring polyphenolic phytoalexin that is present in abundance in many fruits, such as grapes, as well as in wine affects metastasis in breast cancer. Lee’s, [41] study was the first to report that oral administration of resveratrol inhibited the metastasis and growth of *4T1*cancer cells to the lungs in a BALB/c murine model of experimentally induced cancer. Based on these findings, he confirmed that resveratrol may inhibit metastasis by decreasing the activity and expression of MMP-9 a family of enzyme related to tumor invasion and metastasis.

However, despite a number of findings on in vitro anticancer property of tea extracts, there is lack of information on the exact molecular mechanism of anticancer activity of tea. In addition, much work has been carried out using green tea which is mainly consumed in Asia but none on black tea which is the principle tea product from Kenya and also the recently produce purple tea. To further establish why cell viability was reduced in the *4TI*cancer cell line, RNA was extracted from the cells for gene expression profiling to elucidate the underling molecular response of cancer cells to administered tea.

### Quantification and assessment of mRNA quality

The quality and quantity of total mRNA isolated from the *4TI* cancer cells was determined using a Nanodrop 2000 spectrophotometer. A ratio of the optical absorbance at 260 nm and 280 nm (A_260/280_) was used to evaluate for residual contamination of proteins. The ratios obtained in this study indicated that the isolated RNA was of the desired quality since the values ranged between 1.76 and 2.08 (Table 2).

**Table 2:** The concentration and quality of mRNA as determined based on absorbance at 260 nm and 280 nm using a Nanodrop spectrophotometer

#	Sample ID	Nucleic acid conc. (ng/μl)	Unit	A260	A280	260/280	MID^a^
1	GT-B	234.3	ng/μl	7.101	3.406	2.08	13
2	CC-B	279.1	ng/μl	8.457	4.119	2.05	14
3	D-B	36.5	ng/μl	1.107	0.598	1.85	15
4	PT-C	94.9	ng/μl	2.876	1.513	1.9	16
5	GT-D	130.3	ng/μl	3.948	2.11	1.87	17
6	BT-C	66.2	ng/μl	2.008	1.022	1.96	18
7	BT-D	88.1	ng/μl	2.67	1.412	1.89	19
8	PT-A	181.7	ng/μl	5.507	2.749	2	20
9	PT-D	80.3	ng/μl	2.432	1.192	2.04	21
10	GT-A	38.7	ng/μl	1.172	0.579	2.02	22
11	CC-C	12.6	ng/μl	0.382	0.189	1.76	23

^a^Multiplex Identifier (MID) stands for multiplex identifier which is a 10 nucleotide identifier sequence for differentiation of multiplex sequence data sets

**Fig. 6:**
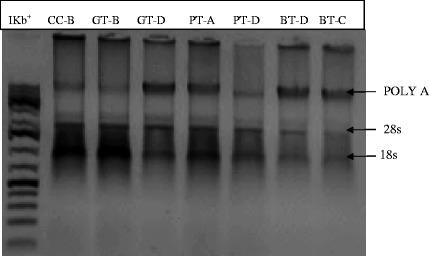
Gel electrophoresis image of mRNA from cells treated with the representative tea samples

The intactness of the mRNA was determined by agarose gel electrophoris. The electrophoresed mRNA formed a smear with two faint bands which indicated that it was not degraded and therefore the extraction procedure had been successful in delivering intact mRNA (Fig. 6). An intact mammalian poly (A) + RNA appears as a smear sized from 0.1 to 4-7 kb or more with faint 28S and 18S rRNA bands.

### cDNA synthesis

**Fig. 7:**
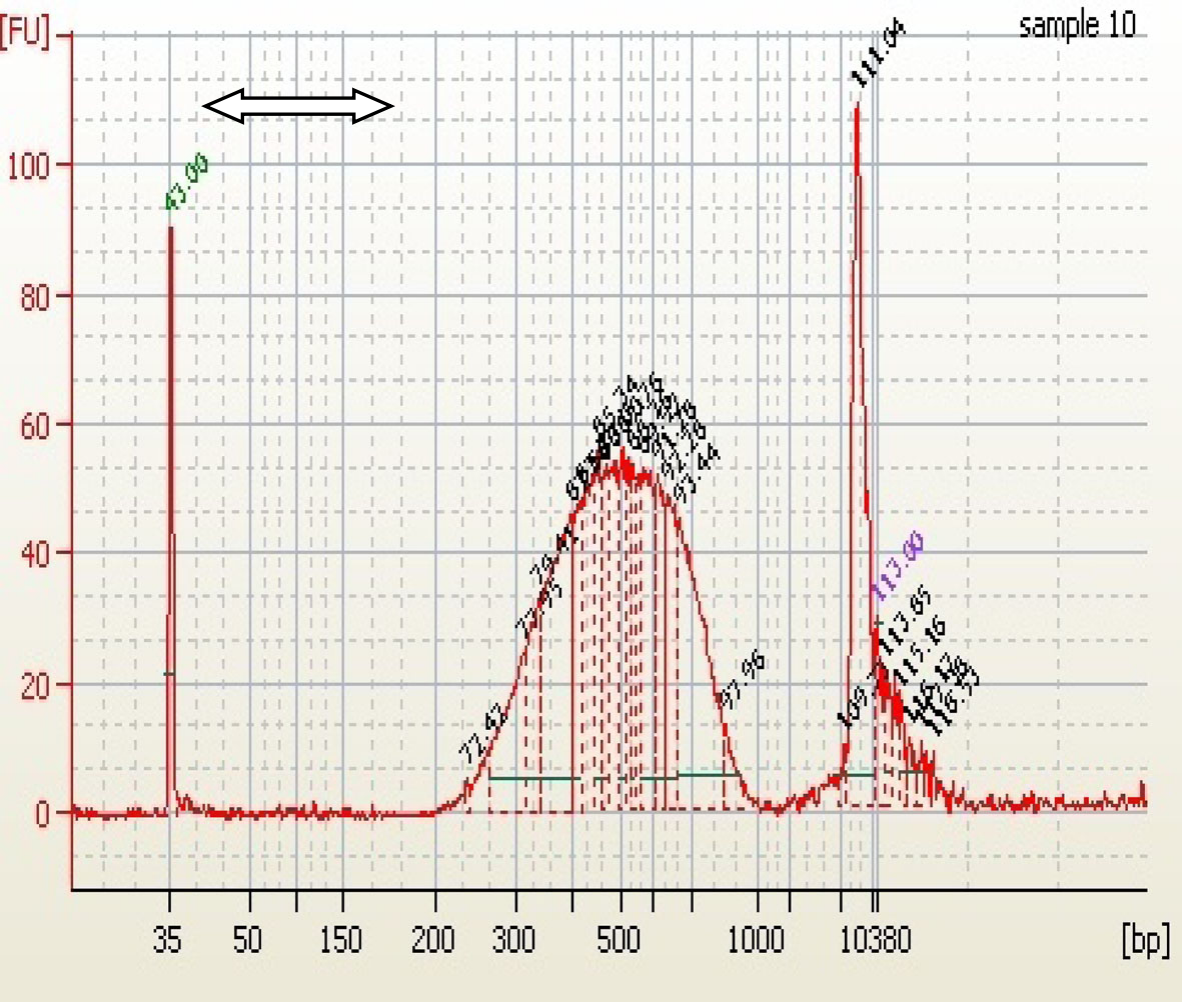
Representative images indicating the size distribution and quality of cDNA library assessed on an Agilent bioanalyzerhigh sensitivity DNA chip. The cDNA produced a peak between 600 and 1200 bp as shown by the arrow

**Fig. 8:**
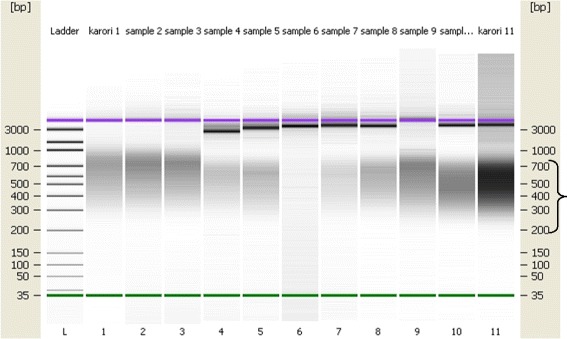
Gel-like image of the cDNA library samples as run on an Agilent bioanalyzer high sensitivity DNA chip. The samples produced a smear that ranged from 0.2 kb to 7 kb as shown in the arrow

cDNA libraries were synthesized from the isolated mRNA and the quality of the cDNA synthesized assessed using a DNA chip. A peak which ranged between 600 and 1200 bp was produced (Fig. 7). A gel like image presentation of the same also produced a thick band at the same fragment length as Fig. 8 which was within the required range for good quality cDNA.

### De novo sequencing of the *4TI* coding transcriptome

**Fig. 9:**
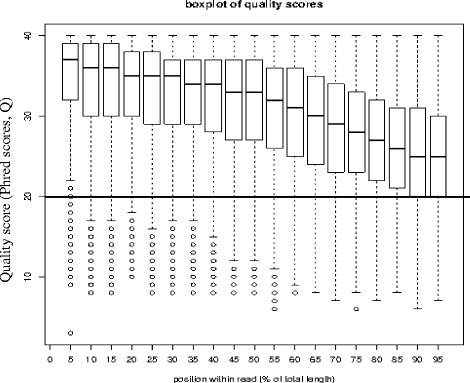
Box plot showing quality scores of trimmed sequences

After the removal of low quality reads, adaptors and sequences less than 50 bp, the quality checks using FastQc showed that all the sequences had a Phred-like quality scores greater than Q20 level (an error probability of 0.01) (Fig. 9). This meant that there was minimal chance of a base being called incorrectly.

### Functional annotation

Mapping of the sequences, processed based on FastQC report, onto the reference mouse genome (*Mus musculus* strain C57BL/6 J) and subsequent differential gene expression analyses revealed green, black and purple tea extracts specific responsive genes in the *4TI* cancer cells. All the extracts, described earlier in the cytotoxicity assay, inhibited cell proliferation relative to the control, as revealed by differential expression of apoptosis related gene family in the transcriptomes. Apoptosis is a critical defense against hyperproliferation of cancer [42] with the results potentially indicative of reduced cell viability with ensuing metabolic processes potentially precipitating apoptosis and necrosis. This is further underpinned by significant induction of caspases namely *casp8* and *casp8AP2* in this study (Table 3) which are established executioners of apoptosis cascades. Since caspases are encoded by cysteine-aspartic acid and act as mediators of apoptosis [43], their significant up regulation in black, green and purple tea treated cell-lines, relative to control indicates induction of apoptosis which is a vital step in fighting cancer manifestations. These finding are in concert with and shed light on potential mechanism of the anti-cancer properties of tea polyphenols, as apoptosis related to induction of *casp8* gene initiates the apoptotic pathway [44–46].

**Table 3:** Transcriptome analysis of genes affected by green, black and purples teas

Gene ID	Green tea	Black tea	Purple tea
*Casp8*	*p* ≤ 0.008, F = 625.98 *R* = 83.87	*p* ≤ 0.007 F = 703.49 *R* = 24.30	*p* ≤ 0.02 F = 246.72 *R* = 59.58
*Casp8ap2*	*p* ≤ 0.03 F = 13.05 *R* = 27.46	*p* ≤ 0.03 F = 60.98 *R* = 11.97	*p* ≤ 0.05 F = 43.51 *R* = 15.71
*Casp3*	*p* ≤ 0.005 F = 15.82 *R* = 15.21	*p* ≤ 0.08 F = 9.4 *R* = 9.27	*p* ≤ 0.05 F = 15.38 *R* = 12.69
*Casp6*	*p* ≤ 0.06 F = 9.956 *R* = 26.306	*p* ≤ 0.02 F = 93.96 *R* = 100.01	*p* ≤ 0.05 F = 323.98 *R* = 26.59
*Apopt1*	*p* ≤ 0.03 F = 10.69 *R* = 23.06	*p* ≤ 0.04 F = 1.53 *R* = 16.46	*p* ≤ 0.02 F = 3.19 *R* = 29.12
*Aifm1*	*p* ≤ 0.03 F = 5.65 *R* = 54.76	*p* ≤ 0.03 F = 95.20 *R* = 8.86	*p* ≤ 0.04 F = 36.55 *R* = 2.80
*Aifm2*	*p* ≤ 0.05 F = 33.68 *R* = 27.54	*p* ≤ 0.03 F = 5.65 *R* = 54.76	*p* ≤ 0.03 F = 5.77 *R* = 47.61
*Casp9*	*p* ≤ 0.01 F = 472.34 *R* = 56.78	*p* ≤ 0.02 F = 281.03 *R* = 26.33	*p* ≤ 0.01 F = 426.82 *R* = 35.11

*Legend*; R; Stands for RPKM which are the reads per kilobase per million is a method of normalization that is wizdely used in RNA-seq analysis

F; Stands for a fold change which is an algorithm used to produce biologically meaningful rankings of differentially expressed genes from RNA-seq data

P; This is a probability of obtaining the observed effect under a hypothesis (null). *P* value of less than 0.05 (*p* ≤ 0.05) are considered statistically significant

The possible mechanism through which the green, black and purple tea induced anticancer responses in this study was through the induction of *casp3* and *casp6* genes which indicated potential cleavage of equally induced caspase-8, a component of the extrinsic apoptotic pathway as has been described by Orbest, [47]. Additionally, the significant up regulation of *casp8AP2* in this study suggests that the extrinsic pathway was initiated. This gene has been shown to be an apoptotic protein which interacts with the death-effector domain (DED) of caspase 8, a component of the death inducing signaling complex. Induction of this pathway has been associated with release of cytochrome c, which was putatively activated as evidenced by the significant induction of *Apopt1, Aifm1* and *Aifm2* genes. The *Apopt1* mediate mitochondria-induced cell death through the release of cytochrome c and activation of the caspase cascade [46]. The *Aifm1* and *Aifm2*, induce mitochondria to release apoptogenic proteins, cytochrome c and caspase 9. In this study *Casp9* was significantly up regulated, a clear indication that the process of apoptosis was executed (Table 3).

Most phytochemicals for example curcumin, thesinensin, resveratrol, acecetin and sulforaphane have been shown to work based on this pathway. Curcumin (diferuloylmethane), the yellow pigment in the rhizome of turmeric (*Curcuma longa*) has been shown to induce apoptosis in several types of cancers including prostate, breast, colon and leukemia through the mitochondrial pathway that involvescaspase-8, BID cleavase, cyt-c release and caspase-3 activation. BCL-2 and BCL-XLare critical regulators of curcumin [48]. Thesinensin-A from oolong tea has also been shown to induce apoptosis through the elevation of ROS production, release of cyt-c, followed by the concomitant induction of caspase-9.

Most phytochemicals for example curcumin, thesinensin, resveratrol, acecetin and sulforaphane have been shown to work based on this pathway. Curcumin (diferuloylmethane), the yellow pigment in the rhizome of turmeric (*Curcuma longa*) has been shown to induce apoptosis in several types of cancers including prostate, breast, colon and leukemia through the mitochondrial pathway that involvescaspase-8, BID cleavase, cyt-c release and caspase-3 activation. BCL-2 and BCL-XLare critical regulators of curcumin [48].Thesinensin-A from oolong tea has also been shown to induce apoptosis through the elevation of ROS production, release of cyt-c, followed by the concomitant induction of caspase-9.

Theaflavins (TF-1 and TF-2) from tea have also been demonstrated to be effective on acute T -cell leukemia and cause a rapid induction of caspase-3 but not caspase-1 [49]. Sulforaphane (SFN) from criciferous plants like broccoli, cabbage, cauliflower and their sprouts causes G2/M–phase arrest and increases apoptotic cell fraction in leukemia cells, markedly increasing P_53_ and Bax expression [50]. SNF brings growth inhibition by inhibition of CDK-4 and CyclinD1 resulting in G1/S cycle arrest with a down expression of BCL2 [51]. Styrylpyrone derivatives (SPD) from *Goniothalamus sp.* inhibit proliferation and induce apoptosis in MCF-7 breast cancer cell line by increasing BAX level and Caspase–9 which in turn activates executionercaspase-7 to bring about apoptosis [52]. Resveratrol, a phytoalexin in the red grape skin (*Vitis vinifera*) acts as an estrogen receptor (ER) agonist and suppresses proliferation by ER independent pathway by inducing up regulation of P21 and brings about growth arrest [53]. At high concentrations it inhibits Cyclin-D and CDK4 and upregulates P_53_ and P_21_ to bring about apoptosis. In MCF-7 breast cancer cell line, it activates Caspase-9 and increases BAX, BAK while decreasing BCL-2 and BCL-XL [54]. Acacetin, a flavonoid of *Cirsium rhinoceros* (Compositae), induces the death receptor pathway by activating Caspase-8 pathway both in HepG2 and A549 cells [55].

It is worth noting that the observed action of tea on caspases through the intrinsic and extrinsic pathway may offer valuable information since commonly used drugs in cancer therapy and management operate based on similar mechanisms. For example, the drugs Cisplatin, Doxorubicin and Methothrexate all up regulate the intrinsic pathway [56, 57] while on the other hand the intrinsic pathway is induced by ionizing radiation and also by chemotherapeutic agents [58]. Since conventional radiotherapy and chemotherapy with synthetic drugs evoke severe side effects including immunosuppression and in a majority of cases organ failure, edible phytochemicals such as polyphenols in tea which are inexpensive, effective, readily available and acceptable may offer an alternative approach to caner control and management. This approach has however been constrained by lack of adequate information on the distinct mechanism of action of the tea phytochemicals. The results obtained from this study on tea and apoptosis offer a better understanding of teas mode of action on cancer. It is anticipated that the information will contribute to the body of knowledge that is necessary for the development of safe multi-targeting anticancer drugs from natural dietary phytochemicals. The findings on the action of tea on caspases offers valuable and insightful information since commonly used cancer drugs operate based on similar mechanisms.

## Conclusion

The chemo-preventive effects of green tea, black tea and purple tea extracts in a highly metastatic breast cancer *4TI* cell line in vitro was carried out for the first time in this study*.* Tea was demonstrated to be a chemo-prevention agent with anti-carcinogenic properties that impact on cell populations by reducing, cell viability and proliferation. Mapping of the sequences obtained from the 454 pyrosequencing platform onto the reference mouse genome *Mus musculus* strain C57BL/6 J and subsequent differential gene expression analyses revealed that green, black and purple tea extracts had specific responsive genes in the *4TI* cancer cells. The following genes were differentially expressed; *casp8, casp8ap2, casp3, casp6, apopt1, aifm1, aifm2 and casp9.*The mechanism through which the green, black and purple tea induced anti-cancer responses in this study was through the induction of *caspases* which induced the apoptotic pathway.

### Recommendations

Polyphenols in tea can be extracted and marketed as pharmacological compounds with anti-cancer activity thus expanding the uses of tea. The possible mechanisms of protection by tea polyphenols against mutagens has been reported in this study, however, further research is needed to determine the mechanism of this action in more details, and to explore other beneficial effects that these polyphenols may have, before they can be adopted for therapeutic use. Using edible or edible-like plants compounds for example tea that has active constituent in our daily life could be the best inexpensive choice for protecting us from cancer. There is need to undertake further studies and fully elucidate the plausible mechanisms of tea antimutagenic and anticancer activity. Caspases and apoptotic pathway should be used in investigating plant compounds with anti cancer activity for drug development. We therefore recommend the use of next generation sequencing platforms to investigate the potential health properties of tea polyphenols and to support research focused on discovery of new natural compounds which could help a lot in investigating or discovery new medicinal drugs especially cancer drugs.

## Abbreviations

AMV: Avian myeloblastosisvirus
CTMDR: Center for Traditional Medicine and Drug Research
EGCG: Epigallocatechin Gallate
emPCR: emulsion PCR
ILRI: International Livestock Research Institute
IREC: Institutional Research Ethics Committee
KEMRI: Kenya Medical Research Institute
MID: Multiplex Identifier
MPC: Magnetic particle separator
MTT: 3-[4, 5-dimethylthiazol-2-yl]-2, 5-diphenyl tetrazoliumbromide
SMPs: Streptavidin-coated–magnetic particles
TF: Theaflavins
TRI: Tea Research Institute
